# Bioinformatic tools for microbiome analysis: from raw sequences to biological insights

**DOI:** 10.3389/fmicb.2026.1913362

**Published:** 2026-08-05

**Authors:** Jenna Poelzer, David S. Wishart

**Affiliations:** 1 Department of Biological Sciences, University of Alberta, Edmonton, AB, Canada; 2 Department of Computing Science, University of Alberta, Edmonton, AB, Canada; 3 Department of Laboratory Medicine and Pathology, University of Alberta, Edmonton, AB, Canada; 4 Faculty of Pharmacy and Pharmaceutical Sciences, University of Alberta, Edmonton, AB, Canada

**Keywords:** bioinformatics, metagenomics, microbiome, review, software

## Abstract

The rapid growth of microbiome research has been accompanied by an expanding but fragmented ecosystem of bioinformatic tools. Researchers now face a daunting array of software packages, pipelines, and web platforms spanning every stage of analysis, from quality control and taxonomic profiling to functional annotation and statistical interpretation. While this diversity offers flexibility, it also creates challenges in selecting appropriate tools and integrating them into coherent, reproducible workflows, particularly for researchers without formal computational training. This review presents a practical, workflow-oriented guide to microbiome data analysis, from raw DNA sequence processing to statistical interpretation and biological insight. We evaluate tools based on ease of use, methodological rigor, computational requirements, and community support, with particular attention to the trade-offs between command-line interface and web-based approaches. We cover both amplicon and shotgun metagenomic strategies for taxonomic and functional profiling, discuss reference database selection, and outline key statistical methods, including differential abundance testing and network inference. We also compare integrated platforms and web-based resources that lower barriers for non-computational researchers and discuss best practices for reproducibility and workflow design. Throughout, we highlight emerging technologies, including machine learning methods that are beginning to reshape the field. Overall, this review serves as a practical guide to navigating the microbiome bioinformatics landscape, helping bridge the gap between methodological complexity and the biological questions that drive microbiome research.

## 1 Introduction

Microbiomes, composed of bacteria, archaea, viruses, fungi and protozoa, inhabit a remarkable range of environments, from soils and oceans to plant root systems and the bodies of animals and humans (Whon et al., 2021). Each microbiome is shaped by its local environment and performs essential ecological and biological functions, including nitrogen fixation, carbon and nutrient cycling and pathogen defense in marine, terrestrial, plant-associated and animal-associated ecosystems (Xun et al., 2024).

Among the many microbiome niches studied, the human gut has received particular attention, because of its key roles in metabolism, immune development, and disease susceptibility (Joos et al., 2025). The Human Microbiome Project demonstrated that microbial diversity and abundance vary widely even among healthy individuals, with strong niche specialization both within and between hosts (The Human Microbiome Project Consortium, 2012). The human gut microbiome has been linked to numerous common disorders including inflammatory bowel disease and metabolic syndrome (Joos et al., 2025), driving substantial investment in DNA sequencing technology and analytical methods. As discussed throughout this review, the tools and principles developed for gut microbiome research are broadly transferable, and have been successfully extended to environmental, agricultural, and other host-associated systems.

Regardless of niche being studied, the central questions driving microbiome research remain: “Who is present?” and “What are they doing?” (Liu Y.-X. et al., 2021). Prior to the advent of next-generation sequencing (NGS), these questions were difficult to answer, as culture-based approaches captured only a small fraction of microbial diversity (Walker and Hoyles, 2023). NGS has made answering these questions substantially easier, but transforming sequence data into biological insight still requires bioinformatic tools capable of robust taxonomic and functional profiling.

Two broad strategies are used to generate metagenomic data: amplicon sequencing and shotgun metagenomic sequencing (MGS). Amplicon sequencing uses PCR to selectively amplify a specific genetic region (such as the 16S rRNA gene for bacteria or the ITS regions for fungi) and then sequences only that region to identify and compare organisms within a sample (Liu Y.-X. et al., 2021). This approach is cost-effective and widely available, but its taxonomic resolution is limited, and it provides only indirect or inferred insights into function. In contrast, shotgun MGS sequences all DNA in a sample, enabling direct, high-resolution taxonomic and functional profiling albeit at greater financial and computational cost (Quince et al., 2017).

Both amplicon sequencing and MGS can be performed using short-read and long-read sequencing platforms. Short-read technologies provide high accuracy and cost-efficiency, making them ideal for large cohort studies. Long-read platforms support improved genome assembly, and when applied to MGS data, can provide strain-level resolution and the detection of structural variants. These advantages come with higher error rates and increased computational demands (Eisenhofer et al., 2024; Kim et al., 2024). Consequently, hybrid approaches that combine both short- and long-read data are becoming increasingly common, capitalizing on the strengths of both technologies.

The dramatic decline in DNA sequencing costs over the past decade has led to an exponential increase in data generation, placing greater computational demands at every stage of analysis, from sequence quality control and host-read removal to taxonomic classification and functional annotation (Beghini et al., 2021). At the same time, the rapid expansion of microbiome bioinformatics has produced a fragmented landscape of diverse metagenomic pipelines, databases, and analytical platforms (Liu Y.-X. et al., 2021). Many of these tools require proficiency with command-line interfaces (CLIs), or advanced programming skills in languages such as R or Python. This creates a persistent “accessibility gap” for microbiome researchers with limited formal computational training (Wen et al., 2023).

This review aims to address the accessibility gap by providing practical guidance for selecting and applying appropriate bioinformatics tools across all stages of microbiome analysis and for users with varying levels of computational expertise. Tools are evaluated based on ease of use, methodological rigor, computational requirements, and community support. Our emphasis on new resources, accessibility for both short- and long-read data, and on end-to-end workflow integration distinguishes this review from earlier guides such as Liu Y.-X. et al. (2021) or Bharti and Grimm (2021).

### 1.1 Metagenomic analysis workflow

**Figure 1:**
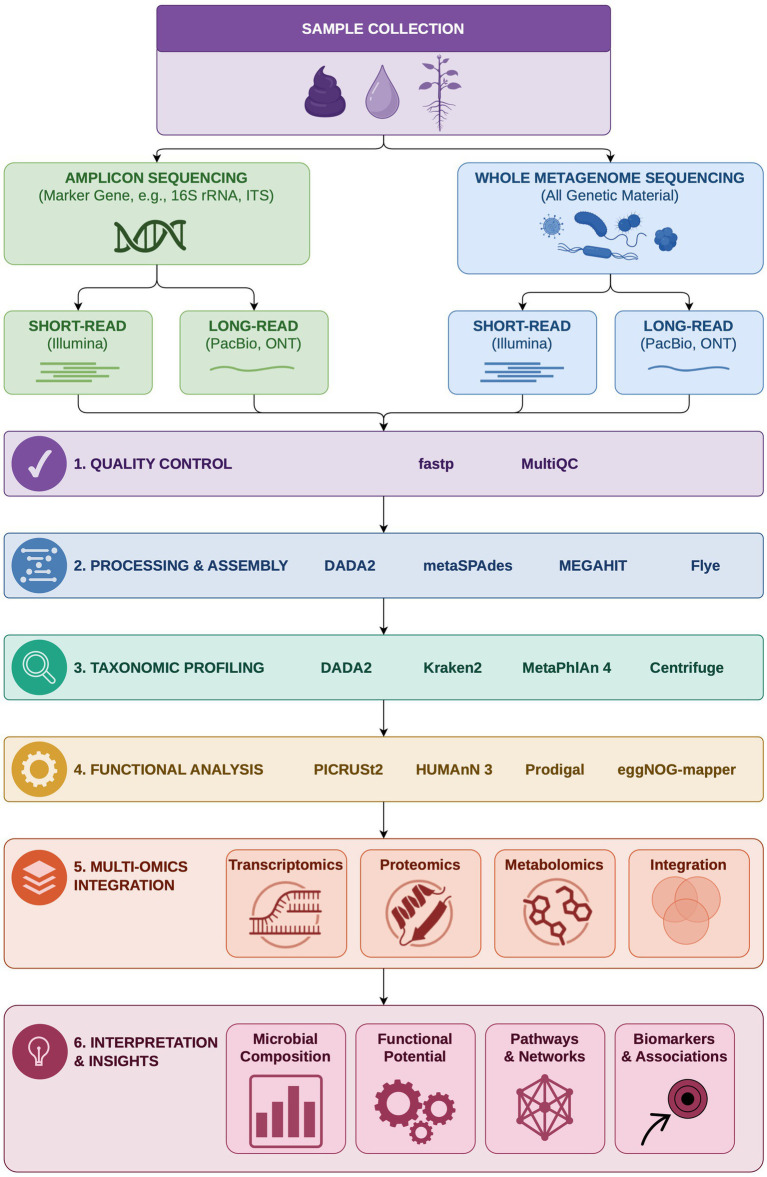
Overview of the whole metagenome sequencing analysis workflow from post-QC reads through sequence processing and assembly, taxonomic profiling, functional analysis, multi-omics integration, and biological interpretation. Recommended tools are grouped by short-read Illumina and long-read sequencing platforms. Text formatting indicates availability: *italics* denote CLI only tools, bold indicates tools available through both CLI and Galaxy, and underlining denotes web-based platforms.

Metagenomic or microbiome analyses involve a series of interconnected workflows that vary depending on the sequencing strategy, including platform choice (long-read versus short-read) and approach (amplicon sequencing versus MGS). Regardless of strategy, all datasets require quality control (QC), filtering, and data pre-processing to remove low-quality reads and technical artifacts. Following these initial steps, analyses diverge into distinct pathways tailored to the study objective, such as taxonomic profiling or functional characterization. These workflows may be implemented using standalone command-line tools, cloud-based web applications, or integrated software suites that bundle multiple functions into a unified pipeline. A broad overview of the workflow, key decision points and representative tools is provided in Figure 1. The following sections describe these workflows in greater detail and critically evaluate the tools and software packages commonly used to implement them.

## 2 Pre-processing and quality control

### 2.1 Raw DNA processing and filtering

**Figure 2:**
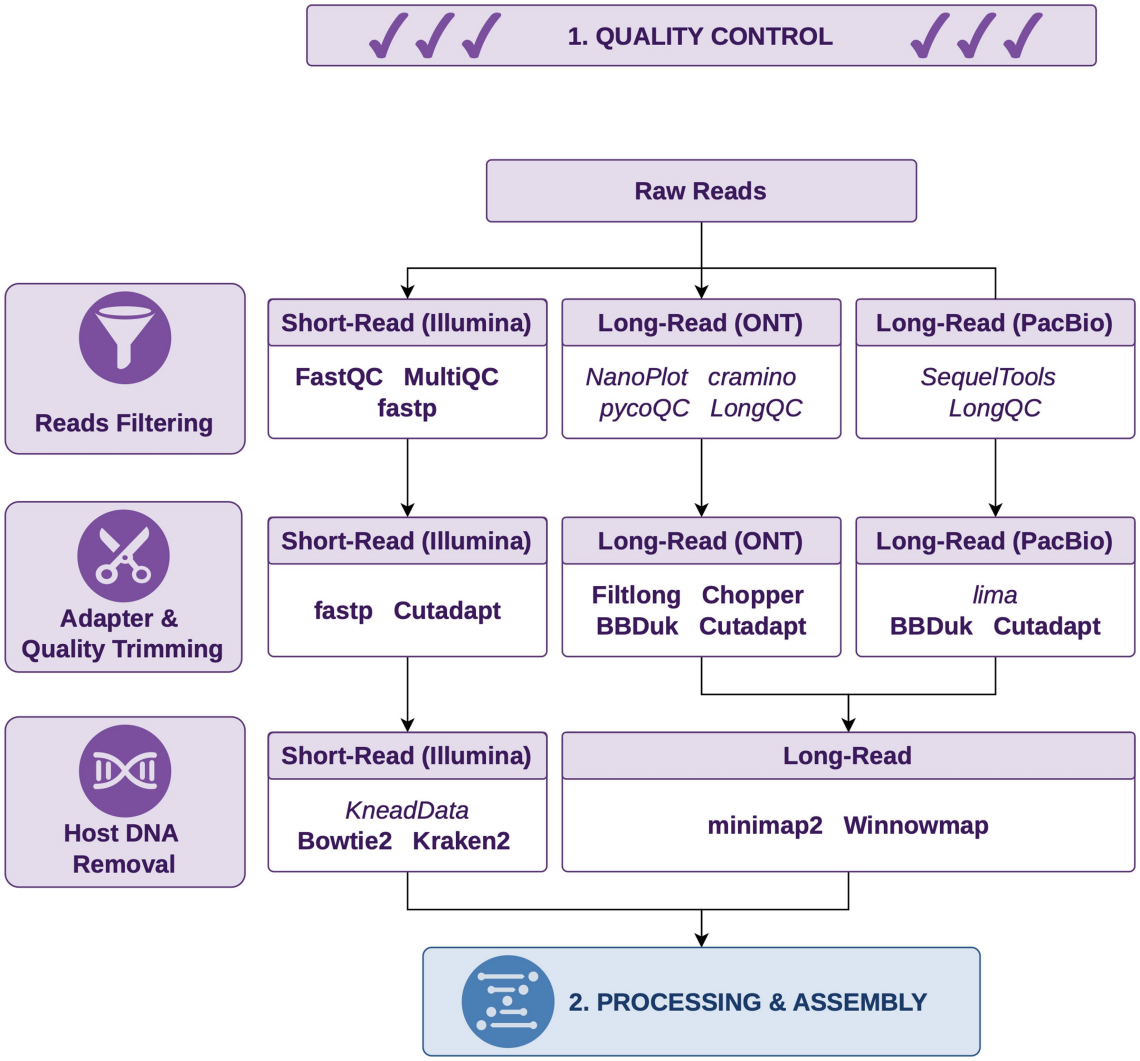
Overview of the complete microbiome analysis workflow, from sample collection through to biological interpretation. Samples from stool, biofluids, or environmental sources can be analyzed using either amplicon sequencing of marker genes (e.g., 16S rRNA or ITS), or whole metagenome sequencing, with both approaches supporting short-read Illumina and long-read PacBio or ONT platforms. The workflow proceeds through six stages with recommended tools highlighted at each step.

All metagenomic sequencing data require careful QC before downstream analysis. Raw reads frequently contain technical artifacts, including low-quality base calls, residual adapters and primers, and contaminating sequences such as host-derived DNA, chimeric PCR products, and index-hopping artifacts. If not removed, these artifacts may bias taxonomic assignments, inflate diversity estimates, and distort functional interpretations (Bokulich et al., 2013). The overall QC workflow is shown in Figure 2.

#### 2.1.1 QC processing for short-read data

**FastQC** (Andrews et al., 2010) remains the standard tool for short-read quality assessment, generating HTML reports that summarize per-base quality scores, GC content, adapter contamination, and overrepresented sequences. For larger projects, **MultiQC** (Ewels et al., 2016) aggregates results across hundreds of samples into a single interactive report, enabling batch-level QC assessment that is not possible with individual **FastQC** reports. Both are available via the command line and the web-based Microbiology Galaxy Lab (**MGL**) (Nasr et al., 2024).

#### 2.1.2 QC processing for long-read data

Long-read QC tools assess read length distributions, error profiles, and sequencing yield rather than cycle-based quality decay. For Oxford Nanopore Technologies (ONT) data, **NanoPlot** (De Coster et al., 2018) provides rich visualizations (length histograms, yield plots, quality-length biplots), while **cramino** (De Coster and Rademakers, 2023) provides rapid post-alignment summarization from BAM or CRAM files. **pycoQC** (Leger and Leonardi, 2019) offers interactive run-level diagnostics for ONT but does not support Pacific Biosciences (PacBio) data. For PacBio Sequel data, **SequelTools** (Hufnagel et al., 2020) performs QC directly from raw BAM files. For multi-platform workflows or studies without a reference genome, **LongQC** (Fukasawa et al., 2020) is the only platform-independent option, supporting PacBio RS-II, Sequel, and ONT MinION data. Many of these tools are available via **MGL**, although **cramino**, **SequelTools**, and **LongQC** remain command-line only.

#### 2.1.3 Filtering and trimming for short-read data

Read filtering and trimming remove low-quality bases, adapter contamination, and unreliable reads, thereby improving alignment accuracy and assembly quality. For short-read data, **fastp** (Chen et al., 2018) is the preferred all-in-one solution, combining quality profiling, automatic adapter detection, polyG trimming, and paired-end base correction while operating 2–5x faster than traditional tool combinations. **Cutadapt** (Martin, 2011) is a well-documented alternative when precise control over adapter sequences or custom trimming logic is required. Both tools are available through the command line and **MGL**.

#### 2.1.4 Filtering and trimming for long-read data

Long-read filtering requires tools designed to accommodate variable read lengths and higher sequencing error rates. For ONT data, **Filtlong** (Wick, 2018) prioritizes high-quality reads based on length and quality metrics, while **Chopper** (De Coster and Rademakers, 2023) provides fast quality and length filtering as a Rust-based successor to NanoFilt (De Coster et al., 2018). **Porechop** (Wick, 2017) handles adapter removal, though it is resource-intensive due to its alignment-based detection. For PacBio HiFi data, **lima** (Pacific Biosciences, 2019) is the standard tool for adapter and barcode removal, though additional filtering is often unnecessary because HiFi reads have already undergone consensus-based error correction. **Cutadapt** supports primer removal across both platforms, while **BBDuk** (Bushnell, 2014) offers platform-independent contaminant screening. For users without command-line experience, **MGL** provides web-based access to many of these tools, although **lima** remains command-line only. Users should be aware that web platforms may impose storage limits on large datasets.

### 2.2 Host DNA removal for shotgun sequence data

MGS samples often contain substantial host-derived DNA, with tissue biopsies reaching 97–99% human reads. High host DNA contamination can increase downstream processing time by nearly 10-fold (Gao et al., 2025; Pereira-Marques et al., 2019). The two dominant algorithmic strategies for host DNA removal are alignment-based and k-mer-based methods. Alignment-based methods map reads to a host reference genome, while k-mer-based methods match short sequence signatures against a database without full alignment, trading accuracy for speed.

#### 2.2.1 Host DNA removal for short-read data

**Bowtie2** (Langmead and Salzberg, 2012) is the most widely used alignment-based tool for short-read host removal, providing accurate detection while only requiring ~3.2 GB of memory for the human genome. It is available via both the command line and **MGL**, although performance declines at very high contamination levels (Gao et al., 2025). **KneadData** (http://huttenhower.sph.harvard.edu/kneaddata), part of the Huttenhower Lab’s **bioBakery** suite (Beghini et al., 2021), wraps **Bowtie2** with quality trimming in a single workflow and includes pre-built databases for human, mouse, and other mammalian hosts. **KneadData** is a convenient option when integrated trimming and removal are preferred, but it requires more RAM (~18 GB) and lacks **MGL** integration. **Kraken2** (Wood et al., 2019) offers a k-mer-based alternative that is substantially faster and less memory-intensive than alignment-based methods (0.3 GB vs. ~18 GB for human genome indexing), making it attractive for large datasets or incomplete host reference genomes. However, this speed comes at the cost of higher false-negative rates, allowing some host reads to escape detection (Gao et al., 2025).

#### 2.2.2 Host DNA removal for long-read data

For long-read data, host removal is best performed with long-read-aware aligners. **Minimap2** (Li, 2018) is the standard choice, efficiently mapping ONT or PacBio reads to a host reference and removing aligned sequences. **Winnowmap** (Jain et al., 2020) improves mapping accuracy in highly repetitive genomic regions by using weighted minimizers rather than repeat masking. However, it is ~15 × slower than **minimap2**, despite having lower memory requirements. **Minimap2** is also available via **MGL**, while **Winnowmap** remains command-line only.

### 2.3 Recommendations

**FastQC** followed by **MultiQC** aggregation is the standard starting point for short-read quality assessment. Long-read QC differs by platform: **NanoPlot** and **cramino** are recommended for ONT visualization and post-alignment summaries, respectively; **SequelTools** is the preferred option for PacBio Sequel data; and **LongQC** is the only platform-independent solution when a reference genome is unavailable.

For short-read trimming, **fastp** is the preferred all-in-one tool, although **Cutadapt** may be advantageous when precise adapter specification is required. For ONT data, **Filtlong** and **Chopper** provide quality and length filtering, while **Porechop** manages adapter removal and **pycoQC** offers interactive run-level diagnostics. For PacBio HiFi data, **lima** within SMRT Link is sufficient, especially as aggressive additional filtering is often unnecessary. Both long-read platforms benefit from **Cutadapt** for primer removal and **BBDuk** for contaminant screening.

Host removal applies only to shotgun sequencing data. For short-read workflows, **Bowtie2** is the best-supported tool, particularly for human stool studies. **Kraken2** is preferable when speed is critical or host reference genomes are incomplete. **KneadData** simplifies read trimming and host removal into a single step albeit with higher RAM requirements. Long-read workflows can typically use **minimap2**, with **Winnowmap** reserved for hosts with highly repetitive genomes such as plants.

## 3 Taxonomic profiling

### 3.1 Amplicon-based profiling approaches

**Figure 3:**
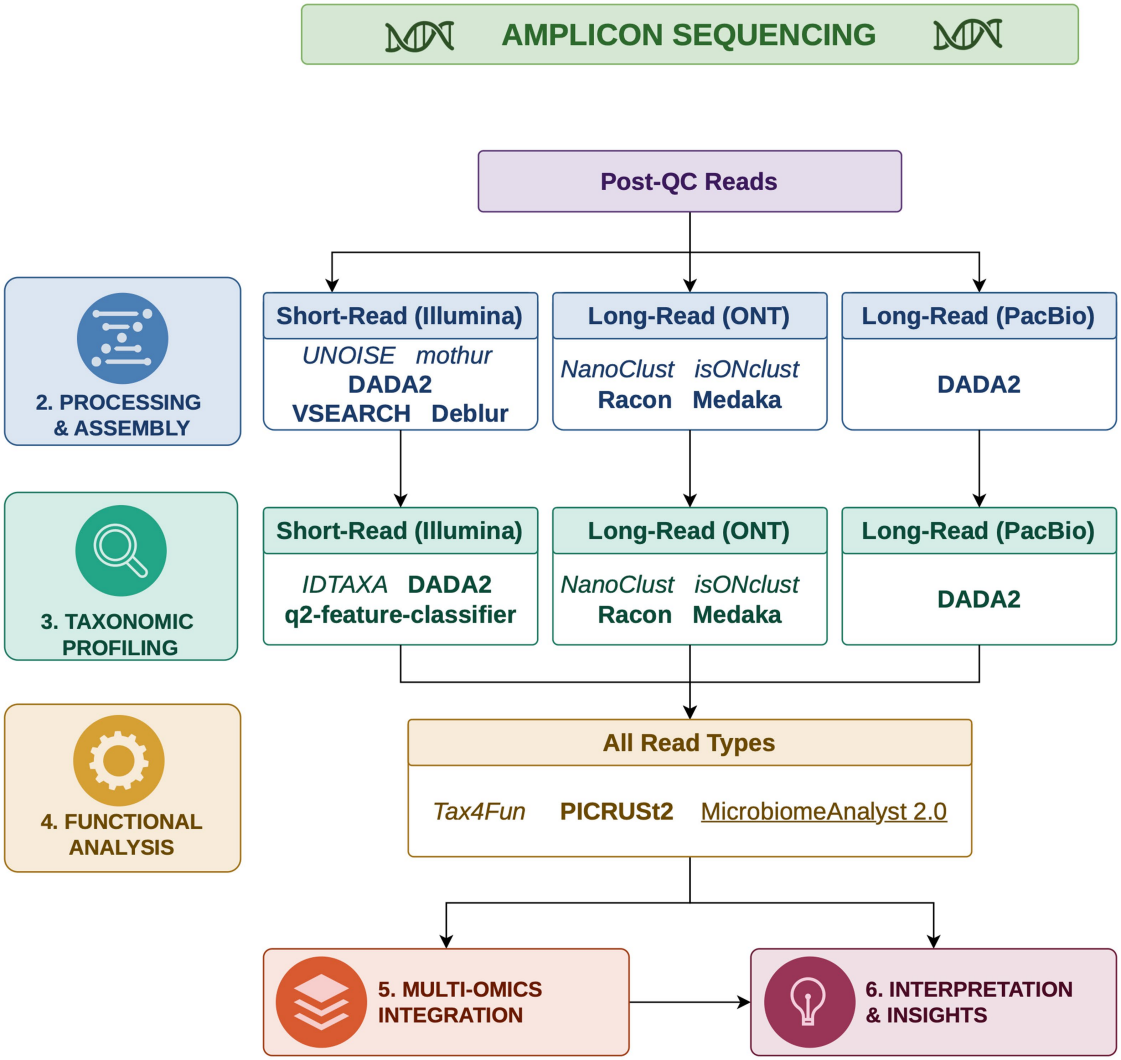
Quality control workflow for raw sequencing reads generated on short-read Illumina, long-read ONT, and long-read PacBio platforms. The three stages shown are reads filtering, adapter and quality trimming, and host DNA removal (not required for amplicon sequencing), with recommended tools listed for each platform. Text formatting indicates availability: Italics denote CLI only tools, bold indicates tools available through both CLI and Galaxy, and underlining denotes web-based platforms.

Amplicon sequencing remains the most widely used method for taxonomic characterization of microbial communities due to its cost-effectiveness, scalability and well-established protocols (Abellan-Schneyder et al., 2021; Liu Y.-X. et al., 2021). Following QC and read cleaning, sequences must be grouped into biologically meaningful units and assigned taxonomic labels to determine community composition (Figure 3). Two major strategies are used for sequence grouping. The first identifies exact biological sequences, known as Amplicon Sequence Variants (ASVs), while the second clusters similar sequences into Operational Taxonomic Units (OTUs). Over the past decade, microbiome research has largely shifted from OTU-based methods to ASV-based approaches because ASVs provide greater taxonomic resolution, reproducibility and comparability across studies (Callahan et al., 2017). By resolving sequences to single-nucleotide differences, ASVs generate consistent labels that can be compared directly between datasets. In contrast, *de novo* OTUs are defined within individual datasets and therefore lack the same level of reproducibility (Callahan et al., 2017). ASVs are also referred to as exact sequence variants (ESVs), zero-radius OTUs (ZOTUs), or sub-OTUs (sOTUs); although all terms describe the same underlying concept of denoised exact sequences. OTU clustering remains in use when comparing to legacy datasets or when broad taxonomic trends are the primary goal rather than fine-scale resolution (Chiarello et al., 2022).

#### 3.1.1 ASV detection and denoising for short-read data

Even after QC filtering, amplicon data often contains errors from PCR and sequencing that, if uncorrected, inflate diversity estimates and produce spurious taxonomic assignments (Callahan et al., 2017). Denoising algorithms address this by learning error models from the data to distinguish true biological variants from noise, yielding ASVs resolved to single-nucleotide precision.

Among available denoising tools, **DADA2** (Callahan et al., 2016) is the most widely adopted for short-read amplicon data. Available as a standalone R package, a **QIIME2** (Bolyen et al., 2019) plugin and through **MGL**, **DADA2** learns sequencing error rates directly from the data to construct a parametric error model. Forward and reverse reads are processed separately before merging and subsequent *de novo* chimera removal. **DADA2** consistently detects more ASVs than competing methods, and generally achieves the lowest error rates (Fares et al., 2025). However, its high sensitivity can occasionally generate spurious variants, particularly in highly diverse environmental samples (Nearing et al., 2018; Nilsen et al., 2024).

Where maximizing sensitivity is less important than avoiding false positives, **Deblur** (Amir et al., 2017) offers a more conservative alternative. Available via the command line and as a **QIIME2** plugin, **Deblur** uses precomputed Illumina error profiles rather than learning error rates from each dataset and processes samples independently using a subtraction-based approach. Prodan et al. (2020) reported perfect specificity with no spurious ASVs, but this accuracy comes at the expense of read retention, often preserving fewer than 50% of input reads compared with more than 70% for other methods. **Deblur** may also be overly conservative for some applications, as it has been reported to remove certain archaeal taxa from highly diverse communities (Nilsen et al., 2024).

For large or high-diversity datasets where computational efficiency is critical, **UNOISE3** (Edgar, 2016a) within the **USEARCH** ecosystem offers a fast alternative. It identifies sequencing errors by comparing sequence abundances assuming that erroneous sequences are less abundant than their true parent sequences. As a result, it is considerably faster than **DADA2**, processing large datasets in hours rather than days. Benchmarking studies have yielded mixed results, with Prodan et al. (2020) finding **UNOISE3** showed the best balance between sensitivity and specificity, while Fares et al. (2025) reported higher error rates in complex mock communities. **VSEARCH** (Rognes et al., 2016) provides a fully open-source alternative with similar functionality and without the memory limitations of **USEARCH**. Although **USEARCH** v12 was released as open source in 2024, it offers reduced functionality relative to earlier versions (Zhou et al., 2024). **UNOISE3** remains command-line only, whereas **VSEARCH** is integrated into both **QIIME2** and **MGL**.

#### 3.1.2 ASV detection and denoising for long-read data

Long-read amplicon sequencing from platforms such as PacBio and ONT relies on a distinct set of denoising and clustering tools compared to short-read Illumina data, owing to fundamental differences in read length and error structure. Short-read data have low, predominantly substitution-based error rates that can be accurately modeled probabilistically, whereas long-read data, especially from ONT, have more complex error profiles including insertions and deletions. This makes direct ASV inference unreliable without prior error correction (Callahan et al., 2019; Karst et al., 2021). Consequently, long-read workflows typically adopt a cluster-then-consensus strategy, grouping similar reads and polishing them to reconstruct the true biological sequence. Short-read workflows follow an error-model-first paradigm that separates true sequences from noise without clustering (Rodríguez-Pérez et al., 2021).

For PacBio HiFi data, reads are generated by SMRT Link using circular consensus sequencing (CCS). CCS uses the number of sub-reads passing over each position to produce highly accurate consensus bases. **DADA2** can be applied to these reads using a CCS-specific error model, leveraging PacBio’s high per-base accuracy to infer ASVs with single-nucleotide resolution (Callahan et al., 2019). **DADA2** is available as a standalone R package, a **QIIME2** plugin and through **MGL**. For ONT data, dedicated clustering tools are typically required before consensus building. **NanoCLUST** (Rodríguez-Pérez et al., 2021) is an ONT-specific pipeline for classifying full-length 16S rRNA ONT reads. It uses Uniform Manifold Approximation and Projection (UMAP) for unsupervised read clustering, followed by polished-read consensus building and taxonomic classification. **NanoCLUST** is implemented as a Nextflow pipeline and is available only through the command line. Alternatively, **isONclust** (Sahlin and Medvedev, 2020) provides a greedy, quality-aware clustering algorithm for ONT or PacBio reads that accounts for variable error rates within reads. It is available via the command line and Bioconda (The Bioconda Team et al., 2018) but is not integrated into **MGL**.

After clustering, consensus sequences are typically polished to improve accuracy. Tools such as **Racon** (Vaser et al., 2017) generate high-quality consensus sequences from long uncorrected reads using partial order alignment, whereas **Medaka** (Oxford Nanopore Technologies, 2018) applies neural network models trained specifically on ONT data. Both tools are command-line based and lack **MGL** integration. The resulting polished consensus sequences are subsequently treated as OTU or ASV-like units for downstream analysis. More general tools such as **VSEARCH** and **mothur** (Schloss et al., 2009) can also process PacBio data and perform clustering, but they are less specialized for long-read error correction and therefore depend heavily on upstream polishing. Both are available through the command line and **MGL**.

#### 3.1.3 OTU clustering for short-read data

Operational Taxonomic Units group sequences with at least 97% similarity, a threshold historically used as a proxy for species-level classification based on early DNA–DNA hybridization studies of full-length 16S rRNA gene sequences (Schloss et al., 2009). By clustering similar sequences, OTU-based approaches reduce dataset complexity while helping to absorb sequencing errors and natural intraspecies variation.

Among available OTU clustering tools, **USEARCH** and its **UPARSE** algorithm (Edgar, 2013) were among the first to combine chimera filtering with OTU clustering in a single workflow, substantially reducing spurious OTU generation. As mentioned, **USEARCH** has remained command-line only since its 2024 open-source release. **VSEARCH** is a fully open-source alternative employing a similar centroid-based clustering strategy, while also supporting dereplication and chimera filtering. It is available through Bioconda and is integrated into **QIIME2** and **MGL**. **Mothur** uses a distance-matrix approach, is well-established, and is **MGL**-compatible, though it is memory-intensive for large datasets (The Galaxy Community et al., 2022).

Despite their historical importance, OTU-based methods have several well-recognized limitations. Studies suggest that species-level classification often requires thresholds closer to 99% for full-length 16S rRNA sequences and 100% for shorter regions such as V4 (Edgar, 2018). Furthermore, clustering results can vary depending on dataset composition and the targeted variable region, reducing reproducibility across studies (Callahan et al., 2017; Johnson et al., 2019).

#### 3.1.4 Taxonomic assignment for amplicon data

Following sequence organization into OTUs or ASVs, taxonomic labels are assigned by comparing sequences to curated reference databases (discussed in the Reference Databases section). Several classification approaches are available, each balancing speed, accuracy, and ease of use differently. The **RDP Classifier** (Wang et al., 2007; Wang and Cole, 2024) pioneered the use of naive Bayesian classification for 16S rRNA gene sequences, providing confidence scores at each taxonomic rank to help assess assignment reliability. It is fast, lightweight, and suitable for large datasets on standard hardware. The **RDP Classifier** is available as a CLI tool, an R package, and integrated into **DADA2**, **QIIME2**, and **MGL**. Accuracy is generally high at phylum and class levels but decreases at genus and species level, particularly for underrepresented taxa (Almeida et al., 2018).

For greater methodological flexibility, **QIIME2’s q2-feature-classifier** plugin (Bokulich et al., 2018) provides naive Bayes, BLAST-based, and consensus taxonomic classification approaches. It is accessible through the **QIIME2** and web-based interfaces such as **MGL**. Pre-trained classifiers are available for many common databases and primer sets, eliminating the need for computationally intensive model training. When feasible, training classifiers on the specific hypervariable region targeted in a study can substantially improve taxonomic accuracy (Werner et al., 2012).

**SINTAX** (Edgar, 2016b), implemented in **VSEARCH** and available through both the command line and **MGL**, uses k-mer similarity to assign taxonomy with bootstrap confidence scores at each rank without requiring a training step. It offers accuracy comparable to the **RDP Classifier** with a simpler workflow. For datasets expected to contain novel or poorly represented taxa, **IDTAXA** (Murali et al., 2018), implemented in the R package **DECIPHER** (Wright, 2016), may provide greater accuracy by reducing the over-classification errors often observed with naive Bayesian methods. However, this improved performance comes at the cost of speed, and **IDTAXA** is available only through CLIs.

Overall, all major classifiers perform well at higher taxonomic ranks, but accuracy decreases at the genus and species levels, especially for underrepresented taxa. No single method consistently outperforms all others across all datasets and experimental conditions (Hung et al., 2022).

### 3.2 Shotgun metagenomics approaches

**Figure 4:**
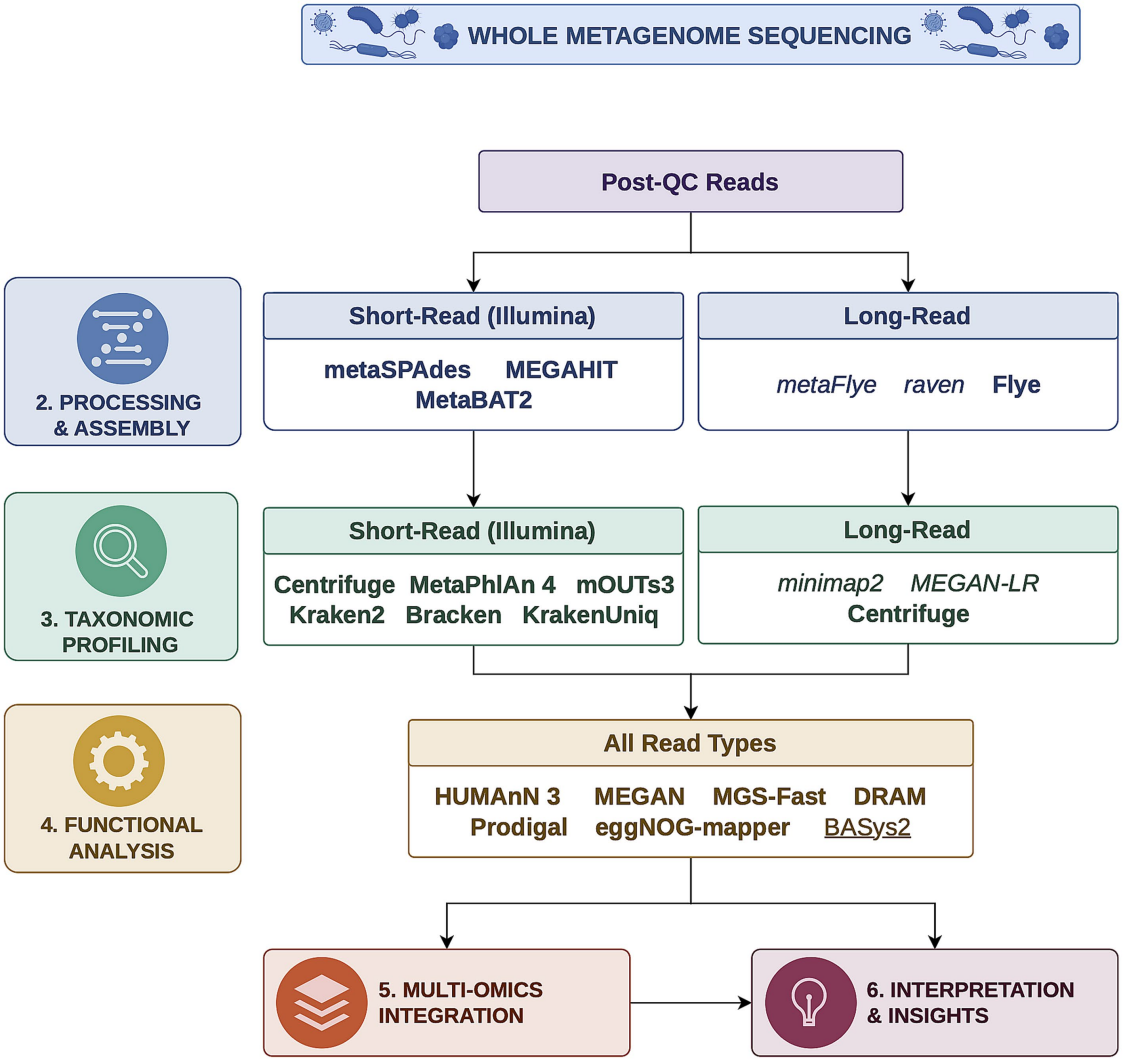
Overview of the amplicon sequencing analysis workflow from post-QC reads through sequence processing, taxonomic profiling, functional analysis, multi-omics integration, and biological interpretation. Tools are grouped by sequencing platform including short-read Illumina, long-read ONT, and long-read PacBio. Text formatting indicates availability: italics denote CLI only tools, bold indicates tools available through both CLI and Galaxy, and underlining denotes web-based platforms.

Unlike amplicon sequencing, shotgun metagenomics is an untargeted approach that sequences all DNA within a sample rather than a specific amplified region. This comprehensive approach enables diverse taxonomic classification strategies, including marker gene–based profiling using curated clade-specific genes, k-mer–based methods for rapid read assignment, and assembly-based approaches that reconstruct longer contigs or genomes before classification (Liu Y.-X. et al., 2021). As with amplicon analyses, tool selection depends on whether the data are short- or long-read (Figure 4).

Short-read data are typically analyzed with k-mer classifiers such as **Kraken2** or marker-based tools like **MetaPhlAn** (Beghini et al., 2021; Blanco-Míguez et al., 2023), optimized for speed and large read volumes. Long-read data are better served by alignment-based tools such as **minimap2** or **MEGAN-LR** (Huson et al., 2018) that accommodate longer sequences and higher indel rates. Reference database choice influences all classification outputs and is discussed in the Reference Databases section below.

#### 3.2.1 Marker gene-based profiling for short-read data

Marker gene-based profilers assign taxonomy to shotgun metagenomic reads by mapping them to curated sets of clade-specific marker genes derived from reference genomes. This approach provides a practical balance between speed, accuracy and computational efficiency without requiring assembly. Widely used tools include **MetaPhlAn 3** (Beghini et al., 2021) and **MetaPhlAn4** (Blanco-Míguez et al., 2023), both part of the **bioBakery** suite. These tools map reads to clade-specific marker genes using **Bowtie2** and are accessible through the command line and Bioconda.

**MetaPhlAn 3** relies exclusively on markers derived from reference genomes, limiting detection to known organisms. **MetaPhlAn 4** extends this framework by incorporating metagenome-assembled genomes (MAGs) organized into species-level genome bins (SGBs). This enables the detection of previously uncharacterized species and increases classification rates, particularly in complex or under-characterized environments (Blanco-Míguez et al., 2023). However, the inclusion of unnamed SGBs (uSGBs) means a portion of classified taxa lack formal species names, which can complicate downstream interpretation and cross-study comparison. Benchmarking studies indicate that **MetaPhlAn 4** does not consistently outperform **MetaPhlAn 3**, with the latter sometimes showing better agreement with established taxonomies (Edwin et al., 2024). Both versions integrate seamlessly with other **bioBakery** tools, including **HUMAnN 3** for functional profiling and **StrainPhlAn** for strain-level analysis.

For environmental or non-human microbiome studies, where reference databases are less complete, **mOTUs3** (Ruscheweyh et al., 2022) provides an attractive alternative. Rather than relying on clade-specific markers, it profiles communities using 10 universal single-copy marker genes, enabling quantification of both known species and previously uncharacterized lineages. Available through the command line and Bioconda, **mOTUs3** has no **MGL** integration. In less-characterized environments, **mOTUs3** and **MetaPhlAn4** outperform k-mer and assembly-based methods, although **mOTUs3** tends to have higher precision at the expense of lower recall (Nickols et al., 2024).

#### 3.2.2 K-mer-based profiling for short-read data

K-mer-based classifiers assign taxonomy by matching fixed-length subsequences (k-mers) from sequencing reads against a pre-indexed reference database. This supports very rapid classification without the need for sequence alignment. **Kraken2** is the most widely adopted k-mer classifier for short-read data. It maps k-mers to the lowest common ancestor of all taxa sharing that k-mer in the reference database. It is substantially faster than alignment-based methods while maintaining comparable accuracy. This is particularly true at the genus level. **Kraken2** supports custom databases built from any collection of reference genomes, though larger databases require more than 100 GB of RAM, limiting their use on standard workstations. Reduced pre-built indexes (8 GB and 16 GB) are available and perform well for many microbiome applications, albeit with some loss of sensitivity (Wood et al., 2019). **Kraken2** is available through the command line, Bioconda, **MGL**, and the **nf-core/taxprofiler** pipeline (Stamouli et al., 2025).

Because **Kraken2** read counts do not account for differences in genome sizes across taxa, it is typically paired with **Bracken** (Lu et al., 2017) for more accurate abundance estimation. **Bracken** uses **Kraken2** classifications and pre-computed k-mer distributions to redistribute reads assigned to higher taxonomic levels to the most probable species or genus, substantially improving quantitative accuracy. Since it builds directly on **Kraken2** databases, no separate database construction is required. **Bracken** is available via the command line, Bioconda, and **MGL**. Using **Kraken2** without **Bracken** for abundance profiling can lead to biased estimates, particularly for higher-level taxa (Lu et al., 2017).

For low-biomass or clinical samples where false positives carry significant consequences, **KrakenUniq** (Breitwieser et al., 2018) provides a more conservative alternative to **Kraken2**. By tracking the number of unique k-mers assigned to each taxon it generates an internal confidence metric that distinguishes true detections from spurious low-coverage matches. Although slower and more memory-intensive than **Kraken2**, it supports database chunking to reduce peak RAM requirements. **KrakenUniq** is available through the command line and Bioconda.

**Centrifuge** (Kim et al., 2016) offers a memory-efficient alternative for taxonomic classification. Its compressed indexing strategy enables the use of very large reference databases, including the full NCBI nt collection, while maintaining modest RAM requirements. This broad coverage makes it particularly useful for studies involving eukaryotic and viral sequences in addition to bacteria. **Centrifuge** is available through the command line, Bioconda, and **MGL**. For highly divergent or novel organisms, **Kaiju** (Menzel et al., 2016) complements nucleotide-based methods by performing translated protein-space searches, allowing classification of taxa that may otherwise be missed.

No single profiling strategy consistently outperforms all others across sample types and study designs (Table 1) (Edwin et al., 2024; Valencia et al., 2024). Consequently, a common best practice is to compare results from complementary approaches, such as **Kraken2/Bracken** and **MetaPhlAn4** or **mOTUs3**. The **nf-core/taxprofiler** pipeline simplifies this process by running multiple classifiers in parallel.

**Table 1:** Tools for shotgun metagenomic taxonomic profiling, grouped by algorithmic strategy: Marker gene-based (low memory; clade-specific databases), k-mer-based (RAM scales with database size), and protein-level classification (improved sensitivity for divergent organisms).

Tool	Read Type	Method	RAM requirements	Interface	Database compatibility
MetaPhlAn3	Short	Marker gene	3 GB	CLI; Bioconda	Built-in marker gene database (RefSeq)
MetaPhlAn4	Short	Marker gene + MAG	3 GB	CLI; Bioconda	Built-in SGB database (RefSeq + MAGs)
mOTUs3	Short	Marker gene	6 GB	CLI; Bioconda	Built-in universal marker gene database
Kraken2	Short	K-mer	8–100 + GB	CLI; Bioconda; MGL	NCBI RefSeq; GTDB; NCBI nt; custom
Bracken	Short	K-mer (post-processing)	Similar to Kraken2	CLI; Bioconda; MGL	Requires existing Kraken2 database
KrakenUniq	Short	K-mer	Similar to Kraken2	CLI; Bioconda	NCBI RefSeq; custom
Centrifuge	Short; Long	K-mer	16 GB for RefSeq	CLI; Bioconda; MGL	NCBI RefSeq; NCBI nt; custom
Kaiju	Short; Long	Protein-level k-mer	10–60 + GB	CLI; Bioconda	NCBI RefSeq; NCBI nr; proGenomes; viruses; fungi
minimap2	Long	Alignment	4–16 GB	CLI; MGL	Any reference genome (FASTA); no built-in DB
MEGAN-LR	Long	LCA (parses minimap2 or LAST alignments)	27–108 GB	GUI; CLI	NCBI; SILVA; KEGG; COG

#### 3.2.3 Alignment-based profiling for long-read data

Alignment-based methods are generally preferred for long-read taxonomic profiling because they better accommodate the higher error rates and more complex error profiles characteristic of long-read sequencing. Unlike short reads, which are highly accurate and well suited to k-mer or marker gene detection, long reads, especially from ONT, contain more insertions, deletions, and context-dependent errors that disrupt exact sequence matching (Delahaye and Nicolas, 2021). Instead, alignment-based methods assign taxonomy by mapping reads directly to reference databases and identifying the best matches (Marić et al., 2024).

Tools such as **minimap2**, **MEGAN-LR**, and **Centrifuge** offer different implementations of this strategy. **Minimap2** is a command-line aligner optimized for long, error-prone reads and is widely regarded as the fastest option. It typically completes large datasets in hours with relatively modest memory requirements (~4–16 GB). However, it performs alignment only and must be paired with downstream tools such as **MEGAN-LR** or **MetaMaps** (Dilthey et al., 2019) for taxonomic classification. **MEGAN-LR** takes alignment outputs from programs such as **minimap2** or **LAST** (Kiełbasa et al., 2011) and assigns taxonomy using a lowest-common-ancestor framework. **MEGAN-LR** provides powerful visualization capabilities through a user-friendly graphical interface, but is substantially slower and more memory-intensive than **minimap2**, often requiring 27–108 GB of RAM for large datasets.

**Centrifuge** occupies a middle ground between these approaches. Using a compressed index of reference genomes, it performs rapid classification with lower memory requirements than **MEGAN-LR** while avoiding the full computational cost of traditional alignment workflows. Although originally developed for short-read data, it has been successfully adapted for long-read applications, albeit with some loss of sensitivity for highly error-prone reads.

#### 3.2.4 Assembly-based approaches for short-read data

Because read-based profiling tools cannot identify organisms absent from reference databases, assembly-based approaches reconstruct longer contiguous sequences (contigs) and draft genomes directly from sequencing reads, enabling the recovery of novel organisms and genes. This workflow consists of three steps: assembly, binning of contigs into putative genomes, and quality assessment of the resulting metagenome-assembled genomes (MAGs). For assembly, **MEGAHIT** (Li et al., 2015) is valued for its speed and memory efficiency (typically 5–12 GB RAM), making it well-suited for large-scale studies. In contrast, **metaSPAdes** (Nurk et al., 2017) generally produces longer contigs and higher quality assemblies and is preferred when computational resources allow (Zhang et al., 2023). Both tools are available via Bioconda and **MGL**.

The second step groups assembled contigs into genome bins. **MetaBAT2** (Kang et al., 2019) uses differential coverage and tetranucleotide frequency to cluster contigs and is widely used because of its speed and accuracy. **MaxBin2** (Wu et al., 2016) employs a similar coverage-based strategy and performs well on single-sample datasets, while **CONCOCT** (Alneberg et al., 2014) uses a probabilistic clustering approach that is particularly effective when multiple samples are available. Because each tool has distinct strengths, running all three and integrating results with **DAS Tool** (Sieber et al., 2018), which selects the highest-quality, non-redundant bins, is common practice. All three are accessible via Bioconda and **MGL**.

The final step is MAG quality assessment. **CheckM2** (Chklovski et al., 2023) uses universal marker genes and gradient-boosted decision trees to estimate genome completeness and contamination. Current community standards define high-quality MAGs as those exceeding 90% completeness while containing less than 5% contamination (The Genome Standards Consortium et al., 2017).

#### 3.2.5 Assembly-based approaches for long-read data

Long-read sequence assembly requires tools built for error-prone reads. **MetaFlye** (Kolmogorov et al., 2020) is the preferred assembler for complex metagenomes, extending **Flye’s** (Kolmogorov et al., 2019) repeat graph approach to resolve strain variation and uneven coverage (32–128 GB RAM). **Flye** is faster and slightly less memory-intensive than **MetaFlye**, being more suitable for lower-complexity samples. **Raven** (Vaser and Šikić, 2021) is the most resource-efficient option (~8–32 GB) and assembles in hours, although with reduced contiguity. All are command-line tools accessible via **MGL**.

### 3.3 Reference databases for taxonomic assignment

The choice of reference database markedly influences taxonomic assignments, affecting diversity estimates, community composition, and the proportion of reads classified at the species level (Molano et al., 2024). Amplicon and shotgun workflows require distinct databases, as amplicon methods compare short marker-gene sequences against curated phylogenetic references, while shotgun tools classify reads against genome-scale collections. The major databases for both are summarized below.

#### 3.3.1 Reference databases: amplicon data

**SILVA** (Quast et al., 2012) is the most widely used amplicon database, covering all three domains of life with ~510,000 non-redundant sequences. It provides pre-trained classifiers for **QIIME2** and integrates directly with **DADA2**. **Greengenes2** (McDonald et al., 2024) inserts 16S sequences into a whole-genome phylogeny, enabling comparisons between amplicon and shotgun datasets from the same samples. **RDP** (Cole et al., 2014) offers a lightweight alternative with minimal computational requirements, while **GTDB** (Parks et al., 2026) provides the highest species-level annotation rates (~90%). However, **GTDB-Tk** (Chaumeil et al., 2022) typically requires 50–150 GB of RAM, limiting its use to HPC environments. Database nomenclature also differs. For example, **SILVA** uses *Bacillota* whereas **Greengenes2** retains *Firmicutes*. Consequently, database choice alone can alter diversity metrics and taxon abundances derived from the same dataset (Molano et al., 2024).

For underrepresented environments or non-standard marker genes, general-purpose databases frequently yield low classification rates (Molano et al., 2024). Ecosystem-specific alternatives such as **HOMD** (Dewhirst et al., 2010) for the oral microbiome or **MarRef** (Klemetsen et al., 2018) for marine systems can substantially improve classification accuracy, although they carry an increased misclassification risk for taxa outside their scope. **RESCRIPt** (Robeson et al., 2021), a **QIIME2** tool, supports the creation of custom databases from NCBI and other repositories, including region-specific trimming to reduce classifier size and improve accuracy.

#### 3.3.2 Reference databases: shotgun data

The NCBI **RefSeq**-based **Kraken2** database (Goldfarb et al., 2025) provides a practical balance of coverage and accessibility for human-associated samples, although it requires ~70 GB RAM and 160 GB disk space (Liu et al., 2024). For assembly-based or environmental workflows requiring high species-level resolution, **GTDB** with **GTDB-Tk** is the preferred alternative. When standard databases are insufficient, custom **Kraken2** databases can incorporate locally assembled genomes (Wood et al., 2019). Classification rates can also be improved by combining ecosystem-specific databases, such as **MiDAS** (McIlroy et al., 2017) for wastewater microbiomes, with broader reference collections, thereby balancing specificity and taxonomic coverage (Dueholm et al., 2020).

### 3.4 Recommendations

For short-read amplicon denoising, **DADA2** is the recommended default. **UNOISE3** is faster for large or high-diversity datasets, whereas **Deblur** is preferable when minimizing false positives outweighs sensitivity. PacBio HiFi data are well-served by **DADA2** operating in CCS mode, while ONT workflows require **NanoCLUST** or **isONclust** paired with **Racon** and **Medaka** polishing. When compatibility with legacy studies requires OTU clustering, **VSEARCH** is the preferred open-source solution, while **mothur** serves as a well-established alternative. Whatever approach is used, validating conclusions across multiple denoising methods is recommended (Nearing et al., 2018). For taxonomic assignment, classifiers trained on the specific hypervariable region generally outperform full-length models (Werner et al., 2012), while **IDTAXA** is often advantageous when novel or divergent taxa are expected.

For shotgun taxonomic profiling, no single classifier consistently outperforms all others across sample types (Edwin et al., 2024; Valencia et al., 2024). Consequently, comparing results from complementary approaches such as **MetaPhlAn 4** and **Kraken2/Bracken** are considered best practice. The **nf-core/taxprofiler** pipeline facilitates this by running multiple classifiers in parallel. **MetaPhlAn 4** is preferred for human-associated studies because it integrates directly with **HUMAnN 3**, whereas **mOTUs3** performs better with environmental datasets. **KrakenUniq** is the right choice when minimizing false positives is critical, and **Centrifuge** provides a practical fallback when computational resources are limited. Long-read data are best handled by **minimap2** paired with **MEGAN-LR** for classification.

Assembly-based approaches require more computational resources but enable recovery of organisms absent from reference databases. For short-read metagenomics, **metaSPAdes** produces the highest quality assemblies, whereas **MEGAHIT** is the practical fallback for large datasets. For long-read MGS, **MetaFlye** is the preferred assembler for complex communities, while **Raven** provides a faster, lower-resource alternative and **Flye** is suitable for simpler assemblies. Genome binning is best performed using multiple tools (**MetaBAT2**, **MaxBin2**, and **CONCOCT**), with results consolidated using **DAS Tool**. Resulting MAGs should be assessed with **CheckM2**, and only genomes exceeding 90% completeness with less than 5% contamination should be retained (The Genome Standards Consortium et al., 2017).

Reference database choice is among the most influential decisions in any profiling workflow. **SILVA** is the recommended default for amplicon studies, whereas **Greengenes2** is best when integrating amplicon and shotgun data from the same samples. **GTDB** is preferred when species-level resolution is the priority and HPC resources are available. Shotgun workflows are best served by **RefSeq**-based **Kraken2** databases for human-associated samples, and **GTDB** is favored for environmental or assembly-based work. In underrepresented ecosystems, combining ecosystem-specific and general-purpose databases improves classification rates. Custom amplicon databases can be built with **RESCRIPt**, and database versions should always be reported to ensure reproducibility.

## 4 Functional profiling

Functional profiling determines the metabolic capabilities encoded within a microbial community. Strategies differ fundamentally by data type (Matchado et al., 2024; Mongad et al., 2021): for amplicon data, functional potential is inferred indirectly using tools such as **PICRUSt2** (Douglas et al., 2020); whereas MGS enables direct characterization through read-based mapping [e.g., **HUMAnN 3** (Beghini et al., 2021)] or assembly-based approaches. Shotgun methods provide a more comprehensive view of community function but require greater sequencing depth and computational resources (Li, 2013; Liao et al., 2014). Outputs are typically abundance matrices mapped to standardized functional frameworks, including KEGG Orthology (KO) groups, Enzyme Commission (EC) numbers, COG categories, GO terms, and protein domain models such as Pfam (Shaffer et al., 2020), allowing gene-level data to be aggregated into metabolic pathways as KEGG (Kanehisa, 2000), MetaCyc (Caspi et al., 2020), or other pathway abundance tables (Beghini et al., 2021; Cantalapiedra et al., 2021).

### 4.1 Predictive approaches for functional annotation of amplicon data

Because 16S rRNA amplicon sequencing targets a phylogenetic marker rather than protein-coding genes, functional annotation must be inferred indirectly: ASV or OTU sequences are matched to annotated reference genomes, gene content is assigned to each taxon, and predicted functions are aggregated across the community according to taxon abundance. The result is a predicted rather than a directly observed functional profile.

**PICRUSt2** is currently the most widely used tool for amplicon-based functional profiling. It accepts ASV (or OTU) tables and representative FASTA sequences, places sequences within a reference phylogeny, and uses hidden-state prediction to infer gene family copy numbers. Outputs include per-sample KO, EC number, and MetaCyc pathway abundance tables, along with a nearest-sequenced taxon index (NSTI) score that identifies taxa poorly represented in the reference database. Although GO terms are not generated by default, they are available through the **PICRUSt2-SC** database and can also be derived from KO outputs using the **ggpicrust2** R package (Yang et al., 2023). **PICRUSt2** can run from the command line, as a **QIIME2** plugin (q2-picrust2), or through **Nephele** (Weber et al., 2018), making it widely accessible. Its primary limitation is higher computational requirements than the original **PICRUSt** due to the phylogenetic placement step.

**Tax4Fun2** (Wemheuer et al., 2020) uses nearest-neighbor genome matching against **SILVA**-annotated RefSeq genomes rather than phylogenetic placement. Implemented as a lightweight R package, it is well-suited for R-based workflows and can incorporate habitat-specific genomes for non-gut environments. A notable feature is its ability to estimate functional redundancy as a proxy for community resilience. **MicFunPred** (Mongad et al., 2021) takes a more conservative approach by predicting only core genes present in ≥50% of genomes within a genus, reducing false-positive inflation. It also uniquely reports KO, EC, Pfam, TIGRFAM, and COG annotations simultaneously.

### 4.2 Functional analysis with shotgun metagenomics data

MGS bypasses indirect inference by sequencing all genomic DNA in a sample, enabling direct detection of functional genes. Two main strategies are used: read-based approaches, which map sequencing reads directly to annotated protein databases, and assembly-based approaches, which first reconstruct longer genomic sequences prior to gene prediction and annotation.

#### 4.2.1 Read-based functional profiling

Read-based methods assign functional annotations directly to quality-filtered sequencing reads by comparing them with protein or gene databases, bypassing the need for assembly. Inputs are typically FASTQ files and outputs are gene family or pathway abundance tables. These methods are faster and less memory-intensive than assembly-based workflows, making them well-suited to large cohort studies. However, they cannot recover complete gene sequences or genomic context.

**HUMAnN 3**, part of the **bioBakery 3** suite, is the most widely used tool in this category. It employs a tiered strategy in which reads are first mapped to a pangenome database using **Bowtie2**, after which unmapped reads are searched against UniRef (Suzek et al., 2015) protein families using **DIAMOND** (Buchfink et al., 2021). Pathway abundances are then reconstructed using **MinPath** (Ye and Doak, 2009) with KEGG and MetaCyc references. Outputs include UniRef gene family and MetaCyc pathway abundance tables stratified by contributing species, allowing functions to be linked to specific taxa (Beghini et al., 2021). **HUMAnN 3** is available as a Python command-line tool, and through **MGL**. Its translated search step is computationally intensive, often requiring 8–32 GB of RAM, 50–100 GB of disk space, and runtimes of several hours.

**MEGAN** Community Edition (Huson et al., 2016) can be paired with **DIAMOND** to provide combined taxonomic and functional profiling against KEGG, COG, eggNOG (Huerta-Cepas et al., 2019), and InterPro (Blum et al., 2025) annotations. Its interactive GUI makes it particularly useful when data exploration and visualization are priorities. **MGS-Fast** (Brown et al., 2019) is a lightweight alternative that annotates reads against microbial gene catalogs using stringent matching criteria. While it lacks the breadth of **HUMAnN 3**, it offers substantially faster runtimes and is available as a Docker container and through **MGL**.

#### 4.2.2 Assembly-based functional annotation

Assembly-based workflows reconstruct contigs from sequencing reads (see Section 3.2.4), then predict and annotate protein-coding genes. The standard workflow follows five steps (Quince et al., 2017): sequence assembly, gene prediction, functional annotation, read mapping back to predicted genes, and gene quantification. Gene counts are normalized using TPM (Transcripts Per Million) for cross-sample comparisons (Wagner et al., 2012). Importantly, annotation scores and e-values reflect assignment confidence rather than gene abundance; abundance estimates are derived entirely from read mapping.

**Prodigal** (Hyatt et al., 2010) is the standard for gene prediction. Operating in a self-training mode, it adapts to the GC content and codon usage of the input sequences without requiring a reference genome. Its metagenomic (“anonymous”) mode is optimized for fragmented contigs and is both fast and memory-efficient. For functional annotation, **eggNOG-mapper** v2 (Cantalapiedra et al., 2021) assigns KO, COG, GO, EC, and Pfam domains via orthology transfer from the **eggNOG** database. Multiple search backends (**DIAMOND**, **HMMER3**, **MMseqs2**) allow users to balance speed and sensitivity. **Prokka** (Seemann, 2014) and its successor **Bakta** (Schwengers et al., 2021) offer integrated gene prediction and annotation workflows for individual MAGs, searching databases such as UniProt, Pfam, and TIGRFAM and producing GFF3/GenBank outputs. **Prokka** is also available through **MGL**. **DRAM** (Shaffer et al., 2020) integrates annotations across multiple databases (KEGG, Pfam, dbCAN, UniRef) and generates pathway completeness summaries, making it particularly useful for MAG-level characterization. However, it requires substantial disk space (≥50 GB) and is best suited for HPC environments. **Prodigal** and **DRAM** are command-line only, whereas **eggNOG-mapper** and **Bakta** also offer web servers for smaller datasets.

For users without command-line experience, **BASys2** (Poelzer et al., 2025) provides a web-based platform for annotating individual MAGs. In addition to GO and COG annotations, it reports operon structure, subcellular localization, metabolic reactions, pathway associations, whole-metabolome annotations and **AlphaFold2**-derived structural proteomics (Jumper et al., 2021), providing more comprehensive outputs than **Prokka** or **Bakta**. A Docker image is available for local batch processing.

#### 4.2.3 Assembly-based gene abundance quantification

Gene abundance is estimated by mapping reads back to predicted gene sequences using **Bowtie2** (also available via **MGL**) or **BWA-MEM** (Li, 2013) (preferred for long-read data). Resulting alignments are stored as BAM files via **SAMtools** (Li et al., 2009). Gene counts can be generated with **featureCounts** (Liao et al., 2014) or **HTSeq** (Anders et al., 2015). At a metagenomic scale, **featureCounts** is considerably faster and is the recommended default, whereas **HTSeq** offers greater flexibility for custom Python-based workflows. Many of these tools are available via **MGL**, although **BWA-MEM** is command-line only.

#### 4.2.4 Pathway reconstruction

Pathway reconstruction converts annotated gene families into organized metabolic pathways, transforming gene profiles into functional insights about community metabolism. In read-based pipelines such as **HUMAnN 3**, this process is performed internally via **MinPath**, which identifies the minimum set of pathways needed to explain observed gene functions. It can reduce false positive pathway assignments by up to 40% compared to naive mapping. Although bulk KEGG data requires a paid subscription, most tools provide compatible mapping files that circumvent this limitation.

For assembly-based analyses, **DRAM** integrates annotation and pathway reconstruction, generating metabolic module completeness summaries and cross-genome heatmaps from MAGs or contigs. However, it requires substantial computational resources (≥50 GB disk) and is best suited to HPC environments. **KEGG Mapper** (Kanehisa, 2000) offers a free web-based alternative for exploratory visualization of KO lists within KEGG pathway diagrams and requires no installation.

### 4.3 Recommendations

Functional inference from amplicon data is inherently indirect, and results should be treated as hypothesis-generating rather than direct measurements. **PICRUSt2** is the recommended default given its active development, **QIIME2** integration, and strong benchmark performance (Douglas et al., 2020; Matchado et al., 2024). **Tax4Fun2** may be preferable for non-gut environments where habitat-specific reference genomes are available.

For shotgun metagenomics, **HUMAnN 3** is the standard read-based profiling tool, offering species-stratified pathway abundances and seamless integration with the **bioBakery** suite. **MEGAN** Community Edition is a strong alternative for non-human datasets or when interactive visualization is prioritized, whereas **MGS-Fast** is well suited to situations where speed is critical or web-based access through **MGL** is preferred.

For assembly-based workflows, **Prodigal** remains the standard for gene prediction, and is commonly paired with **eggNOG-mapper** for KO, COG, GO, and Pfam assignment. **DRAM** is preferred for MAG-level pathway reconstruction and completeness assessment on HPC infrastructure, while **BASys2** provides a web-based alternative for individual MAGs. Regardless of the workflow chosen, pathway identifiers (e.g., **KEGG** or **MetaCyc**) should be selected early and used consistently throughout the analysis (Liu Y.-X. et al., 2021).

## 5 Statistical analysis and visualization

As shown in Figure 1, downstream analysis begins once taxonomic and functional profiles are generated from amplicon or MGS data. At this stage, researchers address questions related to community diversity, differential abundance, predictive modeling, and co-occurrence, using a range of univariate and multivariate statistical methods. The R environment (Ihaka and Gentleman, 1996) is the dominant platform for these analyses, with many widely used metagenomic tools implemented within it. For a comprehensive R-based workflow, Wen et al. (2023) provides practical guidance on best practices across major microbiome analysis packages. However, for those without R experience, this stage presents a steeper learning curve than earlier workflow stages, where GUI-based options are more prevalent. This accessibility gap should be considered when selecting analytical tools. A summary of the major tools for differential abundance, diversity, and network analysis is provided in Table 2.

**Table 2:** Statistical analysis tools for microbiome data, including differential abundance methods, diversity and ordination frameworks, and co-occurrence network approaches.

Tool	Method	Handles Compositionality	Supports Complex Designs	Interface
phyloseq	Data management; ordination (PCoA; NMDS; CCA)	—	Moderate	R / Bioconductor
vegan	Community ecology; PERMANOVA; ANOSIM; ordination	—	Yes (covariates; unbalanced)	R / CRAN
microbiome	Extensions to phyloseq (diversity; compositional transforms)	—	Moderate	R / Bioconductor
microeco	All-in-one: diversity; DAA; networks; functional prediction	—	Yes	R / CRAN
DESeq2	Negative binomial with shrinkage estimation	Partial (assumes most taxa unchanged)	Limited	R / Bioconductor; MGL
ANCOM-BC / ANCOM-BC2	Sampling fraction bias correction	Yes	Yes (BC2: multi-group; ordered; covariates)	R / Bioconductor; QIIME2; MGL
ALDEx2	Monte Carlo Dirichlet sampling	Yes	Limited	R/Bioconductor; QIIME2
LEfSe	Kruskal-Wallis + linear discriminant analysis effect size	No	No	CLI (Python); MGL
MaAsLin2	Generalized linear/mixed models	Partial	Yes (covariates; repeated measures; longitudinal)	R/Bioconductor; CLI
SPIEC-EASI	Graphical lasso/neighborhood selection	Yes	Moderate	R/Bioconductor

Compositionality column indicates whether the method was designed to account for the relative nature of sequencing data.

### 5.1 Compositionality and normalization

A central challenge in microbiome data analysis is compositionality, which arises because sequencing data are inherently relative: counts represent proportions constrained by a fixed total number of reads per sample (Gloor et al., 2017). Sequencing measures the relative distribution of taxa rather than absolute abundance and because all counts must sum to a constant total, the abundance of each taxon is mathematically dependent on all others. Consequently, an increase in one taxon can appear to decrease others, even when their true abundances remain unchanged. This can lead to spurious correlations and misleading statistical inferences if compositionality is ignored (Gloor et al., 2017; Weiss et al., 2017). Differences in sequencing depth across samples further complicate interpretation, as higher read counts can artificially inflate the apparent abundance of taxa (Paulson et al., 2013).

Normalization mitigates these issues by making samples more comparable. Rarefaction (subsampling to a common depth) is widely used for diversity analysis but discards data and is generally inappropriate for differential abundance testing (McMurdie and Holmes, 2014). Alternative methods include total sum scaling (TSS) and cumulative sum scaling (CSS) as implemented in **metagenomeSeq** (Paulson et al., 2013), and RNA-seq-derived methods such as variance-stabilizing transformation in **DESeq2** (Love et al., 2014) and trimmed mean of M-values (TMM) in **edgeR** (Robinson and Oshlack, 2010). Each method involves trade-offs and no single approach is optimal for all datasets. Consequently, normalization methods should always be reported, as different choices can yield substantially different results from the same dataset (Nearing et al., 2022).

### 5.2 General statistical tests for microbiome data

Many microbiome questions can be addressed using standard non-parametric tests applied to summary metrics. Because diversity measures are often non-normally distributed, the Wilcoxon rank-sum test is commonly used for two-group comparisons, the Kruskal-Wallis test for three or more groups, and Spearman correlation for associations with continuous variables (Liu Y.-X. et al., 2021). These methods are implemented in widely used microbiome platforms, including **QIIME2** and **phyloseq** (McMurdie and Holmes, 2013).

### 5.3 Diversity analysis

Diversity analysis is typically the first statistical step following generation of a taxonomic abundance table. It provides a high-level view of microbial community structure by quantifying within-sample diversity (alpha diversity) and between-sample differences (beta diversity). Alpha diversity captures the richness (number of taxa) and evenness (distribution of abundances) within individual samples, using metrics such as Shannon, Simpson, or observed species counts (Cassol et al., 2025). Beta diversity, in contrast, assesses differences among samples or experimental groups using distance metrics such as Bray-Curtis, Jaccard, or UniFrac (Kers and Saccenti, 2022; Lozupone and Knight, 2005). Together, these analyses reveal broad ecological patterns, identify group-level differences, and guide subsequent statistical testing.

The **vegan** package (Oksanen et al., 2022) is the most established R toolkit for ecological diversity analysis and serves as the foundation for many microbiome workflows. It supports diversity indices, dissimilarity matrices, and permutation-based tests including PERMANOVA, which is widely used for beta diversity testing as it accommodates multiple covariates and unbalanced study designs. **Phyloseq** provides a more integrated framework that stores the count table, taxonomy, sample metadata, and phylogenetic trees within a single object, reducing the risk of data mismatches. It imports data directly from **QIIME2**, **mothur**, and BIOM files, and returns **ggplot2** objects for flexible visualization. Together, **vegan** and **phyloseq** cover the core diversity analysis needs of most microbiome studies.

### 5.4 Differential abundance analysis

Differential abundance analysis (DAA) seeks to identify taxa whose abundances differ significantly between groups. However, microbiome count data pose several statistical challenges: they are compositional (relative rather than absolute), zero-inflated (many taxa are absent in some samples), and overdispersed (variance exceeds the mean). Consequently, no single method has emerged as a universal standard. In a large benchmark comparing 14 DAA methods across 38 16S datasets, Nearing et al. (2022) found substantial variation in both the number and identity of significant taxa identified by different tools. Accordingly, the authors recommend applying at least two methods and prioritizing taxa consistently identified across approaches.

**DESeq2**, originally developed for RNA-seq, is widely used for microbiome DAA due to its familiarity and ease of integration into existing workflows. It models counts using a negative binomial distribution, but its normalization assumptions can be violated in microbiome datasets, where many taxa are differentially abundant, potentially inflating false discovery rates (FDR) (Lin and Peddada, 2020). It remains a practical option within R/Bioconductor or **MGL**.

**ANCOM-BC** (Lin and Peddada, 2020) was designed specifically for microbiome data and is generally preferred when compositional bias is a major concern. It estimates and corrects sample-specific sampling fractions using a linear regression framework, providing more reliable FDR control than many RNA-seq-derived methods. It also reports confidence intervals aiding interpretation of effect sizes. **ANCOM-BC2** (Lin and Peddada, 2024) extends this framework to multigroup comparisons, ordered categories (such as disease stages), and covariate adjustment. Both versions are available in R/Bioconductor, **QIIME2’s q2-composition** plugin, and **MGL**.

**ALDEx2** (Fernandes et al., 2014) takes a more conservative approach and is particularly useful where minimizing false positives is a priority. It uses Monte Carlo Dirichlet sampling to model compositional uncertainty before statistical testing, typically identifying fewer significant taxa but producing more reproducible results across datasets (Nearing et al., 2022). **ALDEx2** is available in R/Bioconductor and as a **QIIME2** plugin called **q2-aldex2**.

**LEfSe** (Segata et al., 2011) combines Kruskal-Wallis testing with linear discriminant analysis to identify and rank potential biomarkers. Because it relies on effect size thresholding rather than formal FDR correction, results may be less consistent across studies (Nearing et al., 2022). Its main advantages are accessibility (available via **MGL** without R) and intuitive cladogram visualizations.

**MaAsLin2** (Mallick et al., 2021) is particularly well suited for complex study designs. Using generalized linear and mixed models, it can account for multiple covariates, repeated measures, and longitudinal data within a single framework. This flexibility makes it especially valuable when controlling confounding factors such as batch effects and clinical variables. Available as both an R/Bioconductor package and a command-line tool, **MaAsLin2** also generates publication-ready visualizations alongside statistical results.

### 5.5 Supervised classification of microbiome data

Supervised classification builds predictive models that distinguish sample groups (e.g., disease vs. healthy) based on microbial composition. Unlike differential abundance analysis, it is best suited for prediction and sample stratification, but requires labeled training data and rigorous validation to avoid overfitting (Nearing et al., 2022). Models trained on one cohort frequently generalize poorly to independent datasets due to batch effects and compositional variability (Gloor et al., 2017).

Random forest is the most widely used classifier in microbiome research because it handles high-dimensional sparse data, is robust to correlated features, and generates interpretable feature importance scores (Liaw and Wiener, 2002; Liu Y.-X. et al., 2021). The **q2-sample-classifier** plugin in **QIIME2** integrates **scikit-learn** (Pedregosa et al., 2011) with built-in cross-validation and feature importance reporting, while **caret** (Kuhn, 2008) provides similar functionality in R. For users without programming experience, **MicrobiomeAnalyst 2.0** (Lu et al., 2023) and **WEGAN** (Sykes et al., 2025) offer GUI support for random forest and other classifiers.

### 5.6 Network analysis

Network analysis is widely used to identify patterns of microbial co-occurrence and potential interactions that are not evident from abundance tables alone. By modeling relationships between taxa, it can reveal ecological dynamics such as competition and mutualism, as well as highly connected taxa that may influence community stability or host health. However, microbiome data are inherently compositional, meaning changes in one taxon affect the observed abundance of others. Consequently, standard Pearson or Spearman correlations can generate spurious associations and should generally be avoided (Fabbrini et al., 2023).

SPIEC-EASI (SParse InversE Covariance Estimation for Ecological Association Inference) (Kurtz et al., 2015), implemented in the R package **SpiecEasi**, is the most widely used compositionally aware network inference method. It uses neighborhood selection or graphical LASSO (Least Absolute Shrinkage and Selection Operator) to infer sparse conditional dependency networks, and performs well on typical 16S datasets. **FlashWeave** (Tackmann et al., 2019) is a scalable alternative for large or heterogeneous datasets. Implemented in Julia, it can incorporate metadata alongside taxa as network nodes, helping identify microbes most closely associated with a phenotype. However, its conservative treatment of structural zeros may reduce power in smaller datasets. For comparing network structure between groups, **NetCoMi** (Peschel et al., 2021) provides a flexible R framework supporting multiple inference methods (including SPIEC-EASI) and enables statistical comparison of network properties such as modularity and hub taxa. Network visualization can be performed in **Cytoscape** (Shannon et al., 2003), a GUI platform that requires no programming experience.

A key limitation of all network approaches is that inferred edges represent statistical associations rather than direct biological interactions, and may arise from indirect effects or unmeasured confounders. Consequently, co-occurrence networks should be regarded as hypothesis-generating rather than definitive evidence of interaction.

### 5.7 Recommendations

**phyloseq** is the recommended starting point for diversity analysis, because it provides a unified data structure that minimizes downstream formatting issues. It is best paired with **vegan** for permutation-based beta diversity testing and **ggplot2** for visualization (Wickham, 2016).

No single method is optimal for all differential abundance analyses. **ANCOM-BC2** is the preferred primary method in most workflows because of its compositional awareness, robust FDR control, and support for complex study designs. **MaAsLin2** is often a better choice for studies involving repeated measures or multiple covariates. Biological conclusions should be restricted to taxa identified by at least two independent methods, with **ALDEx2** serving as a useful conservative complement (Nearing et al., 2022).

Random forest remains the recommended default classifier for supervised microbiome analysis. K-fold cross-validation is essential, and models should be validated on an independent test set before drawing biological conclusions (Gloor et al., 2017).

**SPIEC-EASI** is the recommended starting point for co-occurrence network inference. **NetCoMi** can be added when comparing network structure between groups, and **Cytoscape** is well-suited for visualization. Because inferred edges represent statistical associations rather than direct biological interactions, network results should be treated as hypothesis-generating.

## 6 Integrated data analysis platforms and webservers

Microbiome analysis involves multiple stages, including quality control, taxonomic profiling, functional annotation, statistical testing, and visualization. These steps often rely on tools with different data formats, programming environments, and computational requirements (Liu Y.-X. et al., 2021). Managing these steps independently increases complexity, reduces reproducibility, and creates opportunities for error (Wratten et al., 2021). Integrated platforms address these challenges by bundling multiple tools into a unified framework, reducing manual data conversion and streamlining the path from raw data to biological interpretation.

Integrated platforms generally fall into three categories: (1) R packages that consolidate statistical analyses within a common data structure and programming environment (Wen et al., 2023); (2) command-line pipelines that automate multi-step workflows through reproducible, containerized execution; and (3) web-based platforms that provide GUIs, requiring little or no programming experience. The optimal choice depends on the user’s computational expertise, study complexity, and available infrastructure.

### 6.1 Integrated R packages

Traditional R workflows often require coordinating multiple packages with different data structures, increasing complexity and the risk of errors. Several integrated frameworks simplify this process. **Phyloseq** is the most widely adopted and serves as the *de facto* data backbone for many R-based microbiome packages. It stores the OTU/ASV table, taxonomy, phylogenetic tree, and sample metadata within a single object, allowing seamless integration with downstream packages. Alternatively, **microeco** (Liu C. et al., 2021) offers a more comprehensive all-in-one option that supports diversity analysis, differential abundance testing, network analysis, and functional prediction within a unified environment. A detailed workflow is available from Liu et al. (2025).

### 6.2 Command-line pipeline frameworks

Integrated command-line pipelines automate multi-step microbiome workflows, reducing errors from manual data handling and improving reproducibility by standardizing software versions and parameters. These pipelines rely on two complementary technologies. Workflow managers such as **Nextflow** (Di Tommaso et al., 2017) and **Snakemake** (Köster and Rahmann, 2012) define and execute analysis steps, manage job scheduling, and scale seamlessly from laptops to HPC clusters. Containerization tools such as **Docker** (Merkel, 2014) and **Apptainer** (Kurtzer et al., 2017) package software and dependencies into portable images, ensuring consistent execution across computing systems. **Docker** is widely used on local and cloud systems, while **Apptainer** is preferred on shared HPC clusters because it does not require administrator privileges. Package managers such as **Bioconda** and **Bioconductor** (Huber et al., 2015) further support reproducibility through version-controlled software installation.

**QIIME2** is the most widely adopted end-to-end framework for amplicon analysis and is increasingly used for shotgun workflows. Its plugin architecture integrates hundreds of methods, while built-in provenance tracking enhances reproducibility. **QIIME2** is accessible via Conda or **MGL** for GUI-based access. For shotgun metagenomics, **bioBakery** is the leading workflow suite, integrating **KneadData** (QC and host removal), **MetaPhlAn 4** (taxonomic profiling), **HUMAnN 3** (functional profiling), and **StrainPhlAn** (strain-level analysis) into a unified pipeline that supports parallel execution on local systems or HPC clusters.

**Meteor2** (Ghozlane et al., 2025) is an integrated command-line workflow installed through **pip** or **conda**. It delivers taxonomic, functional, and strain-level profiling from a single unified pipeline and database. It maps reads against compact, ecosystem-specific gene catalogues rather than assembling them, and draws functional capacity from KEGG, CAZyme, and antibiotic resistance gene annotations. Its fast mode runs in minutes on roughly 5 GB of RAM, so a standard laptop suffices and no HPC is required. In benchmarks it matched or outperformed the **bioBakery** tools **MetaPhlAn 4** and **HUMAnN 3** on low-abundance species and functional accuracy.

**SqueezeMeta** (Tamames et al., 2019) automates assembly-based workflows, including assembly, gene prediction, functional annotation (KEGG, COG, Pfam), read mapping, abundance estimation, and binning. It generates sample-comparable functional abundance tables with minimum user intervention but requires substantial computational resources (64–128 + GB RAM) and runtimes ranging from hours to days for large datasets.

**nf-core/mag** (Krakau et al., 2022) is a **Nextflow**-based MGS pipeline that performs quality control, host removal, assembly, binning, bin refinement, quality assessment, and annotation. It is particularly well suited for reproducible analyses on HPC and cloud environments within the broader nf-core ecosystem (Ewels et al., 2020). The same community also maintains **nf-core/ampliseq** (Straub et al., 2020), a standardized, containerized pipeline for amplicon workflows.

**ATLAS** (Kieser et al., 2020) is a **Snakemake**-based MGS pipeline covering many of the same assembly and annotation steps as **nf-core/mag** but with an emphasis on ease of deployment on both laptops and HPC clusters.

### 6.3 Web-based analysis platforms

Web-based platforms lower the barrier to microbiome analysis by providing GUI-driven workflows that require neither local installation nor programming expertise. However, they may offer less flexibility than command-line tools and can raise data privacy concerns for sensitive datasets. Major platforms are compared in Table 3.

**Table 3:** Integrated web-based platforms for microbiome data analysis.

Platform	Input data	Amplicon support	Shotgun support	Statistical analysis	Functional profiling	Local install option	Data privacy
MicrobiomeAnalyst 2.0	Processed tables, raw amplicon reads	Yes	Yes	Comprehensive	Yes (PICRUSt2, Tax4Fun2)	Yes (R package)	Cloud (Google/McGill)
METAGENassist	Processed tables (CSV, QIIME, mothur, MG-RAST, MEGAN, STAMP)	Yes	Limited	Moderate	No	No	Remote server
WEGAN	CSV, TXT count or abundance tables	Yes (post-processing)	Yes (post-processing)	Comprehensive (ordination focus)	No	Yes (Docker)	Remote server
MG-RAST	Raw reads (FASTQ, FASTA)	Yes	Yes	Basic	Yes (SEED, KEGG, COG)	No	Remote server (user-controlled sharing)
Galaxy (microGalaxy)	Raw reads to processed tables	Yes	Yes	Comprehensive	Yes	Yes (self-hosted)	Configurable
Nephele	Raw reads (amplicon and WGS)	Yes	Yes	Basic to moderate	Yes (bioBakery)	No	AWS cloud
Namco	Raw FASTQ or processed OTU/ASV tables	Yes	No	Comprehensive (network focus)	Yes (PICRUSt2)	Yes (Docker)	Remote server
SHAMAN	Raw FASTQ or processed tables	Yes	No	Comprehensive	No	Yes (Docker, conda, R)	Remote server

All platforms provide GUI-driven workflows that eliminate the need for local installation or programming experience.

**MG-RAST** (Meyer et al., 2008) is a web-based platform for automated QC, annotation, and comparative analysis of amplicon, shotgun, and metatranscriptomic datasets. It requires no local infrastructure, provides a RESTful API for programmatic access and serves as a large public repository enabling cross-study comparisons. Its primary limitation is reduced flexibility due to fixed pipeline parameters. **MGL**, built on **Galaxy**, supports 300 + tool suites and 100 + workflows, spanning the full microbiome analysis pipeline. It offers shareable, reproducible workflows and can be deployed locally by institutions, although its extensive options still require thoughtful tool selection. **Nephele** provides free cloud-based amplicon and shotgun workflows through a simple interface, but offers limited customization on its AWS infrastructure, which may be a consideration for sensitive data.

**MicrobiomeAnalyst 2.0** is the most feature-rich platform for downstream microbiome analysis, supporting differential abundance testing, functional profiling, multi-omics integration, cross-study meta-analysis, and even end-to-end 16S analysis from raw reads. In an independent benchmark of eight platforms, it achieved the highest overall performance score (Kan et al., 2026) and is freely accessible without registration. **Namco** (Dietrich et al., 2022) also offers an end-to-end 16S workflow including network and ML analyses, while supporting Docker-based local deployment for sensitive data. **SHAMAN** (Volant et al., 2020) takes a similar end-to-end approach, moving users from raw 16S reads through **DESeq2**-based statistical modelling to interactive visualization. Like **Namco**, it can be installed locally with **Docker** when data privacy is a concern. **METAGENassist** (Arndt et al., 2012) remains a well-established platform for comparative metagenomics from pre-processed abundance tables and performs particularly well for beta diversity analysis (Kan et al., 2026). **WEGAN** exposes the full **vegan** R toolkit through a web interface, providing the most comprehensive ordination capabilities of any platform reviewed here, while also supporting Docker deployment.

### 6.4 Recommendations

For R-based workflows, **phyloseq** remains the recommended foundation, whereas **microeco** is better suited to users seeking a single framework for diversity, differential abundance, and network analyses.

Pipeline selection should be guided by data type and study objectives. **QIIME2** remains the most feature-rich and widely supported framework for amplicon studies, **bioBakery** provides the most integrated solution for read-based MGS, **nf-core/mag** is well-suited to containerized assembly-based workflows, and **SqueezeMeta** is preferable when integrated quantification across co-assembled samples is required.

For researchers without programming experience, **MicrobiomeAnalyst 2.0** offers the most comprehensive single-platform solution for downstream analysis. **Namco** and **WEGAN** are attractive options when data privacy is a concern, while **MGL** and **Nephele** offer user-friendly, end-to-end workflows starting from raw read data. Regardless of platform, workflow definitions, software versions, database versions, and parameter settings should always be documented to ensure reproducibility (Wratten et al., 2021).

## 7 Discussion

Microbiome bioinformatics has matured into a rich but increasingly fragmented ecosystem. Researchers can now profile microbial communities at resolutions ranging from kingdom to strain level (Blanco-Míguez et al., 2023) and link taxonomic composition to metabolic function (Beghini et al., 2021; Douglas et al., 2020). Consequently, the primary challenge is no longer the lack of analytical tools but selecting and applying the most appropriate ones.

Microbiome analysis is inherently a multi-step, decision-dependent process. Choices made at each stage, from database selection and normalization to statistical testing, can substantially influence downstream results. An inappropriate reference database, unsuitable normalization method (Nearing et al., 2022; Weiss et al., 2017), or failure to account for compositionality (Gloor et al., 2017) can lead to misleading conclusions. Consequently, published workflows should be treated as adaptable frameworks rather than fixed protocols (Wratten et al., 2021).

A significant gap remains between analytical sophistication and user accessibility (Wen et al., 2023). Web-based platforms such as **MicrobiomeAnalyst 2.0** and **MGL** have lowered barriers to entry and are well suited for exploratory analysis, but they lack the flexibility required for more complex or large-scale studies. For researchers willing to develop basic programming skills, integrated R frameworks such as **microeco** provide a practical middle ground, offering greater flexibility and reproducibility without the full complexity of custom command-line workflows (Wen et al., 2023).

Reproducibility is a persistent challenge in microbiome research, because differences in tools, parameters, and reference databases can produce markedly different results from the same dataset (Calnan et al., 2024). At a minimum, software versions and database versions, parameter settings, and normalization methods should be reported (Wratten et al., 2021). Wherever possible, analyses should be conducted within containerized environments managed by workflow systems such as **Nextflow** or **Snakemake**. Built-in provenance tracking in **QIIME2** and the containerized workflows maintained by the **nf-core** community (Ewels et al., 2020) provide practical models for reproducible microbiome analysis.

Although microbiome bioinformatics is relatively mature, the field continues to evolve rapidly, and no single review can capture every dimension of that growth. Eukaryotic, viral, and resistome analysis, multi-omics integration, and other biological interpretations are active areas of development that fall outside the scope considered here and are addressed in other publications (Deek et al., 2024; Galeeva et al., 2025; Peng et al., 2021; Thielemann et al., 2022). Long-read sequencing technologies are expanding opportunities for strain-level profiling, full-length 16S classification, and recovery of complete metagenome-assembled genomes (Agustinho et al., 2024). Machine learning methods are also being applied with increasing sophistication and, in some cases, outperforming classical statistical approaches (Nearing et al., 2022). At the same time, large-scale MAG recovery efforts are expanding reference databases (Blanco-Míguez et al., 2023; Parks et al., 2026), improving classification in previously undercharacterized environments. Amid these advances, the key principles remain: researchers must match the method to the question, understand underlying assumptions, and rigorously document every analytical decision (Gloor et al., 2017; Nearing et al., 2022; Wratten et al., 2021).
